# Endoscopic Endonasal Transsphenoidal Resection for Pituitary Apoplexy during the Third Trimester of Pregnancy

**DOI:** 10.1155/2014/397131

**Published:** 2014-01-02

**Authors:** Adesh Tandon, Juan Alzate, Patrick LaSala, Marvin P. Fried

**Affiliations:** ^1^Department of Neurosurgery, Montefiore Medical Center, 3316 Rochambeau Avenue, Bronx, NY 10467, USA; ^2^American Center for Spine and Neurosurgery, Libertyville, IL, USA; ^3^Department of Otorhinolaryngology, Montefiore Medical Center, Bronx, NY, USA

## Abstract

Pituitary apoplexy is an uncommon phenomenon typically characterized by vascular insufficiency or acute hemorrhage into a pituitary adenoma. The overall incidence of pituitary apoplexy ranges between 1 and 25% of all pituitary adenomas. With the widespread use of MRI technology, the diagnosis of asymptomatic intratumoral hemorrhage is closer to 10%. The authors report a case of a 27-year-old female in her 36th week of pregnancy who presented with severe onset headache and acute left-sided vision loss. MRI of the brain revealed a large hemorrhagic mass occupying the sella turcica. The patient underwent an emergent endoscopic endonasal transsphenoidal resection for pituitary apoplexy. Postoperatively, the patient's neurologic deficit resolved. Minimally invasive endoscopic endonasal transsphenoidal resection of pituitary apoplexy can be safely utilized in third trimester pregnant women presenting with acute severe neurologic deficits.

## 1. Introduction

Schloffer and Hochenegg performed the first successful transsphenoidal surgery in 1908 while in Vienna [1]. Soon thereafter, Harvey Cushing perfected the transsphenoidal approach for sellar lesions in 1910, but he later abandoned it secondary to its limited exposure. In the 1960s, the introduction of the operative microscope by Hardy and the advent of fluoroscopy by Giout resulted in the resurgence of the approach [1, 2]. While the introduction of endoscopic techniques in several surgical fields have become the standard of care for many pathologies, only recently, neurosurgeons have become more interested in applying this technology in the management of some unique disease entities. Over the last decade, neurosurgeons have advanced minimally invasive endoscopic techniques in dealing with pituitary lesions. There have been a number of studies comparing more traditional “open” approaches to the sella, with endoscopic assisted and with purely endoscopic surgeries. In most of these studies, there are no statistically significant differences between the three different types of treatments with regards to outcome [3–10]. Herein, we present the case of a 27-year-old pregnant female who developed acute vision loss during her third trimester, secondary to pituitary apoplexy. She successfully underwent a minimally invasive endoscopic endonasal transsphenoidal resection of the pituitary apoplexy.

## 2. Case Report

### 2.1. History and Examination

**Figure 1:**
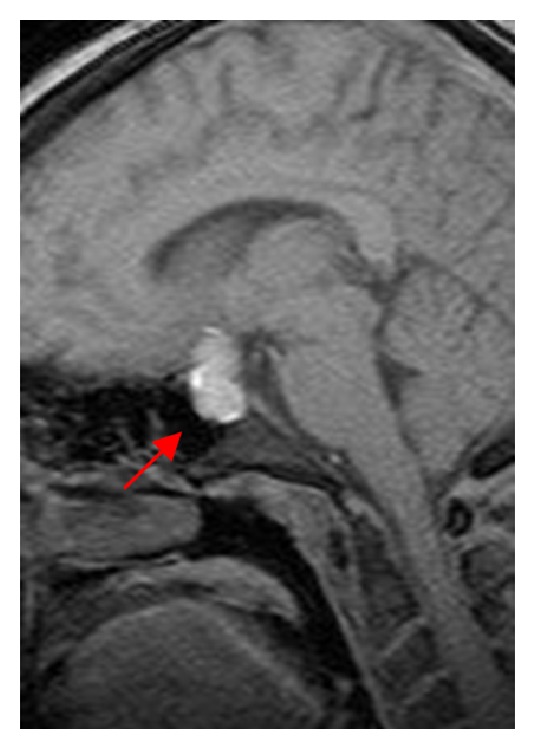
Sagittal T1 MRI of brain without contrast shows area of hyperintensity within the sella and suprasellar regions (red arrow). This likely represents pituitary apoplexy within the known macroadenoma.

This 27-year-old pregnant woman, presented initially at 19 weeks gestation with intermittent headaches, nausea, and vomiting for two months duration. She was admitted to an outside hospital and was diagnosed with a prolactin secreting pituitary adenoma. At that time, the patient was noted to have normal visual acuity in both eyes with no evidence of field cut as well. The patient was treated with bromocriptine therapy given the diagnosis of prolactinoma. At 36-week-gestation, the patient presented to our hospital with severe onset headache and acute vision loss in the left eye. MRI of the brain revealed a suprasellar, hemorrhagic mass measuring approximately 2.1 × 1.3 cm in size with noted optic chiasm compression (Figure 1). On neurological examination, the patient was noted to have decreased visual acuity in the left eye (20/200), with normal vision in the right eye. Visual field testing revealed a severe bitemporal visual field loss. All other cranial nerves remained intact. Given the significant, acute, neurologic deficit noted upon the examination, the decision was made to offer the patient minimally invasive surgical intervention. Prior to the planned surgery, a lengthy discussion with the anesthesia team was conducted with regards to risks to the unborn fetus. A multidisciplinary “team” approach was implemented to ensure the greatest degree of safety for the patient.

### 2.2. Operation and Postoperative Course

**Figure 2:**
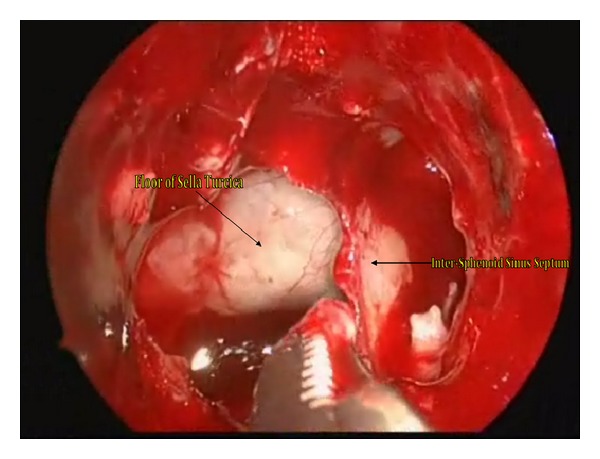
Intraoperative view through the operative endoscope revealing the intersinus septum within the sphenoid sinus and the floor of the sella turcica.

**Figure 3:**
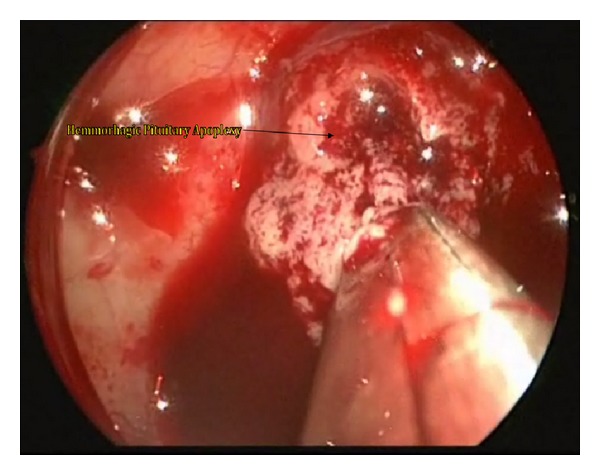
Intraoperative view through the operative endoscope revealing the hemorrhagic pituitary apoplexy being removed in a piece meal fashion.

**Figure 4:**
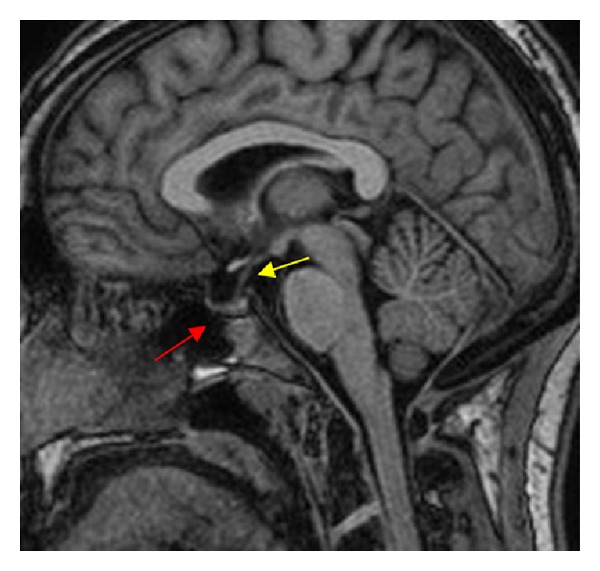
Postoperative sagittal T1 MRI of brain without contrast shows resolution of the prior areas of hyperintensity within the sella turcica (red arrow). The pituitary stalk is now visible (yellow arrow).

The patient was taken to the operating room to undergo an endoscopic endonasal transsphenoidal resection of the pituitary apoplexy. The patient's head was immobilized using a three-pin head fixation device. The operation was performed endoscopically through both nostrils. The left middle turbinate was fractured outward to widen the corridor to the sphenoethmoid recess in order to facilitate the identification of the sphenoid ostium. Bilateral anterior sphenoidectomies were performed extensively with Kerrison rongeurs. The intersinus septum of the sphenoid sinus and sinus mucosa were then removed (Figure 2). The bone in the anterior wall of the sella was notably attenuated. The exposed dura mater was then incised and old blood products extruded under pressure once the dura was opened and an internal decompression of the tumor was performed (Figure 3). The interface between the pseudocapsule of the tumor and normal pituitary gland was identified and dissected with a microdissector and a small ring curette. Once the remaining tumor had been identified in the corners, it was removed under direct endoscopic visualization. Care was taken not to tear the arachnoid membrane to reduce the risk of postoperative cerebrospinal fluid leakage. The entire procedure lasted less than two hours. The patient was transferred to the neurosurgical intensive care unit for postoperative care. On postoperative day one, the patient developed polyuria and increased serum sodium. The patient was treated with desmopressin and liberal intake of water for central diabetes insipidus. The patient's serum sodium stabilized after one day and the central diabetes insipidus remained only transiently. Upon examination, the patient demonstrated rapid visual acuity and visual field improvement. Finally, the patient was transferred to the labor and delivery unit where an elective Cesarean section was done one week after the endoscopic endonasal surgery was concluded. The newborn and the mother remained in stable condition. Postoperative MRI revealed complete excision of the tumor, with no further evidence of optic chiasm compression (Figure 4).

### 2.3. Pathological Examination

Frozen sections revealed mainly blood products with fragments of pituitary gland tissue. The histopathology was consistent with prolactinoma. 

## 3. Discussion

Prolactinomas and nonsecreting adenomas are the most common pituitary tumors. They represent 40% and 39% of all pituitary tumors, respectively [10]. Pituitary adenomas, especially prolactinomas, may evolve as macroadenomas or microadenomas [11]. Clinically significant increases in size occur in approximately 1–5% of all microprolactinomas [12].

During pregnancy, there is a normal increase in the volume and T1 hyperintensity of the anterior pituitary as demonstrated by MRI. This is explained by a relative increase in the number of lactotrophs of the pituitary gland. Normally, lactotrophs comprise from 15 to 20% of the gland. During pregnancy this number may increase to almost 50%, which accounts for the increase in prolactin production [11].

Macroprolactinomas account for the most common pituitary lesion in pregnant women. Approximately 35% of macroprolactinomas enlarge during pregnancy making their medical or surgical management a priority prior to the pregnancy [13]. The initial management of prolactinomas during pregnancy is medical therapy with dopamine agonists. However, in cases of pituitary apoplexy associated with acute optic chiasm or optic nerve compression, the management requires prompt surgical intervention to decompress the optic apparatus [14–18]. Surgical intervention is indicated to prevent permanent vision loss.

Pituitary apoplexy is characterized by the abrupt destruction of pituitary gland tissue secondary to infarction or hemorrhage of the gland itself. This phenomenon is usually more common in macroprolactinomas and in some rare cases may be associated with lymphocytic adenohypophysitis [15, 19]. Clinical features of pituitary apoplexy include severe headache, stiff neck, fever, visual disturbances, and symptoms of adrenal insufficiency accompanied with circulatory shock. Anticipation of this clinical entity and prompt recognition of symptoms may prevent disastrous consequences. Subacute pituitary apoplexy occurs in about 10 to 15% of adenomas, but, in general, clinical symptoms remain mild in pregnant women [13].

Once the pituitary apoplectic event is identified in a pregnant woman, care must be directed to both mother and fetus in a manner designed to optimize the physiologic stability of both. A “team” approach is required, which includes a neurosurgeon, an ICU personal, and an obstetrician [20]. Prompt ICU care and subsequent neurosurgical intervention can lead to improvements in neurological deficits. Traditionally, an open transseptal approach may have been used for resection of the pituitary adenoma, but more recently some neurosurgeons may choose to utilize the endoscopic endonasal approach to the sella turcica.

The endoscopic, endonasal, transsphenoidal surgery has comparable surgical outcomes to conventional microscopic transsphenoidal surgery [3–8, 21–23]. Patients' generally have a quick recovery, short hospital stays, and minimal postoperative discomfort [24]. The two main advantages of the endoscopic approach, when compared with the standard microsurgical operation, arise from the dynamic optical ability afforded by the endoscope and from the absence of a transsphenoidal retractor [14]. The endoscope allows for greater visualization within the sella turcica itself. With the addition of the 30 degree endoscope, surgeons can visualize portions of the pituitary tumor that they may have tried to blindly dissect with the use of the operative microscope. The endoscope also allows for clear delineation of the arachnoid membrane and so may facilitate a lower risk for cerebrospinal fluid leakage. Additionally, with the absence of sphenoidal retractors surgeons gain greater visibility and dexterity of their microsurgical instruments.

Gondim et al. described a case of a patient with a macroadenoma in treatment with bromocriptine that was stopped after the patient became pregnant. In the third trimester, pituitary apoplexy developed requiring surgical treatment. The patient underwent a successful endoscopic endonasal resection of the pituitary apoplexy [16]. The patient reportedly had resolution of her neurologic deficits. More recently, Iuliano and Laws described two cases where pituitary apoplexy developed in pregnant women. Both cases were initially managed conservatively but ultimately required operative intervention to prevent vision loss [25].

Our patient presented with headache and visual loss, with a known history of a pituitary tumor. As suspected, MRI confirmed pituitary apoplexy in this pregnant woman. The patient underwent a minimally invasive, endoscopic, endonasal pituitary apoplexy resection. Postoperatively, the patient's visual disturbances resolved and she was safely able to undergo an elective Cesarean section one week later. 

## 4. Conclusion

Pituitary apoplexy in pregnancy is a rare event. The new advances in endoscopic surgery permit a rapid, minimally invasive treatment, in cases associated with acute optic nerve compression. The endoscopic, endonasal approach is a safe alternative to open cranial surgery for the treatment of pituitary apoplexy in third trimester pregnant women.
